# Supplementary material for: On characterizing gender and locational composition of adult PLHIV in Nigeria: Implications for HIV programming
Source: PLOS Glob Public Health. 2024 Aug 26;4(8):e0002863. doi: 10.1371/journal.pgph.0002863 (PMC11346663; doi:10.1371/journal.pgph.0002863)
Supplement: Attachment — Submitted filename: Review Comments.docx [file pgph.0002863.s001.docx]

**Comments**

**General**

The authors tried to characterize gender and locational composition of adult persons living with HIV by relying on data from the 2018 HIV/AIDS Indicator and Impact Survey.

The following recommendations will best improve the suitability of the manuscript for publication;

The authors indicated that the prevalence of HIV was 1.4%. (1.8% among females and 1.0% among males). This revelation is the most important foundation of the overall study; hence, it needs to be vividly explained in the narrative beneath table 3.

On the prevalence of mother to child transmission, it appears confusing. It is recommended that authors create a separate table to capture the accompanying data with the attendant narrative being explained in detail. The tables appear lumped and clamped together which makes it difficult to locate the findings the authors are pointing to.

Once the authors are interested in providing the locational composition of adult PLHIV, I was expecting to see a GPS map with accompanying coordinates that display such locational composition of adults living with HIV, this will give findings more scientific outlook. So, I recommend authors to incorporate that.

**Specific**

**Introduction**

1. **The statement in Line 14 &15 stated that,** Nigeria is the fourth largest country accounting for HIV prevalence of 1.4% in individuals (15 – 49 15 years), having an approximate of 1.9 million people living with HIV (PLHIV) in 2019 (3,4). The statement is not clear.

**Methodology**

2. **The statement in line 21 “**Publicly available data from the Nigeria HIV/AIDS Indicator and Impact Survey 2018” was it a secondary data that was used? If it is so, it should be captured as such
3. Since the data used in the research was a secondary data, **line 22,23, 24** should be talking about data extraction (ie. How your variables of interest were extracted from the Nigeria HIV/AIDS survey dataset).

**Statistical procedure**

4. Line 18 and 19 stated that, the nonresponse adjustment cells were constructed using the Chi-square Automatic Interaction 19 Detector (CHAID) algorithm. Why this tool?
5. Line 20, we don’t extract prevalence from table and figures. Please tell how the prevalence was calculated.
6. Line 6 stated that, and STATA version 18, and statistical significance was set at 10%.

Please can you justify why 10% significant level?

7. Generally, the data analysis/ statistical procedure is inadequate and should be relook into detail with relevant information.

**Results,**

8. Table1, the age groups are too many, it should be reduced by regrouping them in ten years intervals
9. Table 2, I think you should reconsider putting the p-values for variable that are statistically significant.
