# Supplementary material for: On characterizing gender and locational composition of adult PLHIV in Nigeria: Implications for HIV programming
Source: PLOS Glob Public Health. 2024 Aug 26;4(8):e0002863. doi: 10.1371/journal.pgph.0002863 (PMC11346663; doi:10.1371/journal.pgph.0002863)
Supplement: Attachment — Submitted filename: Review Comments_19022024.docx [file pgph.0002863.s003.docx]

**Reviewer #3**

Thank you for the opportunity given to me to peer review your work. Below is the feedback that I have after a review of your manuscript.

Overall, this manuscript provides a comprehensive overview of the HIV situation in Nigeria. The introduction effectively sets the stage by presenting global and national statistics, highlighting the key populations driving the epidemic, and emphasizing the importance of targeted interventions.

The methodology section is detailed and well-structured, explaining the data source, design, participants, sampling procedure, and statistical procedures.

The ethical considerations section adequately addresses the use of secondary data and emphasizes adherence to ethical guidelines during the original survey. However, considering that data was already approved, the ethical consideration section should be shorter.

RESPONSE: The authors appreciate this comment and have made the ethical considerations shorter.

For the figures depicting the percentage of households with at least one HIV-positive member (Figure 2) and HIV prevalence among participants by state (Figure 3) are informative and help to convey the regional disparities. However, it would be beneficial to include more detailed discussions or interpretations of these figures in the text.

RESPONSE: The authors appreciate this comment and have included more detailed discussions to the discussion section of the manuscript.

Other suggestions for improvement

1. Discussion: While the paper provides a comprehensive set of results, the discussion section could be expanded to provide more in-depth interpretations of the findings. You can discuss the implications of the observed regional and gender disparities and how these insights can inform targeted interventions.

RESPONSE: The authors have further added that the high prevalence among mothers can increase the prevalence of HIV in future.

2. Clarity in Reporting: Some sentences are complex and could be simplified for clarity. Ensure that the language used is accessible to a broad audience, especially if the paper is intended for policymakers or healthcare practitioners.

RESPONSE: The authors have reviewed the discussion again, and simplified some complex words for clarity.

3. Citations: Ensure that all statements and statistics are appropriately cited. While the paper generally provides sources for the data and information presented, a few statements could benefit from more specific citations.

RESPONSE: The authors have reviewed all discussions and added citations where necessary

Reviewer #4: The Manuscript covers secondary data covering 220000 individuals , 2 stage Clustering Sampling technique,, characterising gender ,Location ,Control, with Test ,Treat , Target at 95 % is praise worthy. Many references given on strategy for prevention , The author recommendation Has not been discussed from data analytics on biomedical, behavioural, and structural infrastructure and access to education for prevention of HIV .While awareness of HIV 58% mother were in urban ,29.8 % in rural ,and also when rural detects early(Page 18 L18,19), how rural prevalence more is surprising.

Geo Clustering to be defined by map for urban to claim the density with earlier reviewer recommendation.

NAIIS focus on 2018, But to maintain volatility of data , the trend in HIV updated conditions be mentioned.

The sources of spread of HIV and the rate of change in viral be highlighted for controlling the spread be discussed apart from prevalence . The high risk population has not been identified from ref 3 and 5 though separately mentioned as MTCT as primary measure. the effectiveness of Anti retro viral therapy be mentioned from referred literature. The procedure of Chi-square detection algorithm be explained for non-respondents . Table 2 and Table 3 are Unwieldy , and will be strenuous to researcher to assimilate. Therefore the scope to concise by presenting only the significant factors of p value below 0.05,and mentioning the event of the row that " does have HIV " only .The compliment event -" does not have HIV " - be removed - may be examined.

Page 3, Line 13 ,15 - !,1 million 2018 and 1.9million 2019 - rate of increase be mentioned

RESPONSE: The authors think that the rate of increase cannot be mentioned because the 1.1million in 2018 represents the incidence of HIV in the continent while 1.9 million representing the total number of PLHIV in Nigeria in 2019.

page 4 line 19 - MTCT is primary measure - literature of strategies be elucidated.

RESPONSE: The authors included a paragraph on “risky behavior and practices” immediately after this section. We believe that this covers preventive measures, as suggested by the reviewer.

page 9 Line 5-15-49 years female -1.7% ,Male -0.8%- P vale significance commented

Page 9 - Line 11,12 -women 1.8% and men -1.0 % slightly higher be tested for statistical significance

RESPONSE: The authors have included the p-value

Page 9 Line 21-Anti retro viral therapy-effectiveness of prevention from reference be discussed

RESPONSE: The authors do not seem to fully understand this comment, as there is no “line 21” in page 9.

Page 16 Line 5,6,7,8,- men , women , rural ,urban , before survey and after survey - the figures are to paraphrased in sentence properly

RESPONSE: Apologies, but we do not seem to understand this comment

Page 16 line 23 , 24 - This sentence statistically significant be rephrased to distinguish between rural and urban .

RESPONSE: The statistical significance was tested across both categories (rural and urban). Therefore, it is not possible to distinguish between both groups of individuals.

page 18 Line 3,4,5 - not evidenced by data and recommended for removal

RESPONSE: The authors derived this information from Table 2, and have included this to direct readers to the table.

Page 18 Line 13,14, 15 -Limited access to education ,healthcare ,and resources be mentioned from literature and not evidenced from data .

RESPONSE: The authors were trying to explain (from literature) the reason for the disparity in rural-urban prevalence of HIV

Page 19 Line 4,5 - Reason for urban detection early is delayed due to patient load has not been evidenced with data .

RESPONSE: The authors found this in a previous literature, and only used this to discuss the reasons for the observed rural-urban inequalities.

Overall It is an informative progressive strength with the trend of spread rate that deserves publication.
