# Supplementary material for: On characterizing gender and locational composition of adult PLHIV in Nigeria: Implications for HIV programming
Source: PLOS Glob Public Health. 2024 Aug 26;4(8):e0002863. doi: 10.1371/journal.pgph.0002863 (PMC11346663; doi:10.1371/journal.pgph.0002863)
Supplement: Attachment — Submitted filename: Comments.docx [file pgph.0002863.s004.docx]

Dear Authors,

Firstly, I would like to commend you on the clear and structured presentation of your manuscript. The choice of Excel for data analysis is particularly noteworthy, as it makes your study accessible and replicable for a wide audience. Overall, the paper is well-written and organized efficiently, which enhances the reader's understanding and engagement with your work.

However, I have a few questions and suggestions that I believe could further strengthen your manuscript:

- **Statistical Analysis (Page 8, Lines 2 and 4**): Your choice to employ both the Chi-square test and Fisher's exact test is appropriate for the analysis conducted. However, could you provide more detailed reasoning for the use of these specific tests in the context of your study? Elaborating on this choice would help readers understand the suitability and implications of these tests for your data and research objectives.
- **Ethical Considerations (Line 12):** You mention an ethics and regulatory board in your discussion on ethical considerations. Could you specify which board this refers to earlier in the text? The mention in lines 17 and 18 suggests a specific board, but clarity from the outset would help readers understand the ethical framework guiding your study. Additionally, considering this is a secondary analysis, could you clarify whether any form of Human subjects’ protection course or certification was completed by the research team? This information is crucial for readers to gauge the ethical rigor of your study.
- **Results Section** - Consideration of Rural vs. Urban Households: It is noted that 57% of the households in your study are in rural areas, leaving 43% in urban areas. This slight imbalance prompts a question regarding the potential impact on your statistical analysis. Have you considered applying weights to the data based on this rural-urban distribution? Although the current imbalance may not be significant, exploring this through weighted analysis could offer insights into whether it influences the results. This could be an interesting aspect to consider or discuss, even if only to validate the robustness of your findings.

In closing, I believe these additional details and clarifications could significantly enhance the manuscript's contribution to the field. Your study presents valuable insights, and addressing these points would only strengthen its impact and reliability.

Kind regards,
