# Supplementary material for: On characterizing gender and locational composition of adult PLHIV in Nigeria: Implications for HIV programming
Source: PLOS Glob Public Health. 2024 Aug 26;4(8):e0002863. doi: 10.1371/journal.pgph.0002863 (PMC11346663; doi:10.1371/journal.pgph.0002863)
Supplement: Attachment — Submitted filename: PGPH-D-23-02288_R2_Lazare_review.docx [file pgph.0002863.s005.docx]

**Peer Review manuscript, by Lazare M'BOUNGOU.**

**1. Manuscript number:** PGPH-D-23-02288R2

**2. Article title reviewed:** *On Characterizing Gender and Locational Composition of Adult PLHIV in Nigeria: implications for HIV programming.*

**3. Outputs of the review:**

***3.1. Review of the authors responses in specific comments***

*Overall, the authors have rigorously followed and integrated all of peer reviewers observations, except mainly the following point :*

*Comment 3 : Since the data used in the research was a secondary data, line 22,23, 24 should be talking about data extraction (ie. How your variables of interest were extracted from the Nigeria HIV/AIDS survey dataset).*

*Authors response : The authors did not extract data from the Nigeria HIV/AIDS survey dataset – only the technical report of the survey was assessed and data was manually extracted from it.*

*My conclusion about this point : This response is acceptable. But, the authors must include this additional information in the document as reported here: the technical report of the survey was assessed and data was manually extracted from it.*

***3.2. My (review) comments***

*The manuscript provides a comprehensive overview of the HIV situation in Nigeria. However, in this manuscrit, hepatitis B and C are presented as opportunistic HIV infections (tables 2 and 3). We suggest these infections be considered as HIV co-infections. Regarding HIV opportunistic infections, we recommend to consider tuberculosis.*

*Unfortunately, this important study has not addressed HIV status among TB population due to the association exists between both infections. We know that TB is considered as the most common opportunistic infection among PLWH, and the prevalence HIV among TB population is often high particularly in African countries.*

*The same is true with the key populations (PS, MSM, IDUs, prisoners, etc.), whose prevalence is often higher than the national average in most African countries (Nigeria being no exception) and depending to the place of residence (rural/urban).*

***3.3. Recommandation :***

*It would be beneficial to address HIV status among TB population and among key populations (PS, MSM, IDUs, prisoners, etc.) based on the place of residence according to place of residence, as well as was done with the other groups in tables 2 and 3. Thus, if data are available, authors are invited to complete the tables 2 and 3 with the HIV situation in TB patients and in key populations. If data are not available, both situations should be discussed with evidence and references in the manuscript.*
