# Supplementary material for: On characterizing gender and locational composition of adult PLHIV in Nigeria: Implications for HIV programming
Source: PLOS Glob Public Health. 2024 Aug 26;4(8):e0002863. doi: 10.1371/journal.pgph.0002863 (PMC11346663; doi:10.1371/journal.pgph.0002863)
Supplement: Attachment — Submitted filename: Review Comments_08032024.docx [file pgph.0002863.s006.docx]

**Reviewer #5**

Firstly, I would like to commend the authors on the clear and structured presentation of your manuscript. The choice of Excel for data analysis is particularly noteworthy, as it makes the study accessible and replicable for a wide audience. Overall, the paper is well-written and organized efficiently, which enhances the reader's understanding and engagement with your work.

However, I have a few questions and suggestions that I believe could further strengthen the manuscript:

• Statistical Analysis (Page 8, Lines 2 and 4): The choice to employ both the Chi-square test and Fisher's exact test is appropriate for the analysis conducted. However, could the authors provide more detailed reasoning for the use of these specific tests in the context of your study? Elaborating on this choice would help readers understand the suitability and implications of these tests for the data and research objectives.

RESPONSE: The authors initially added the reason for the adoption of chi-square, which was stated as: “In order to assess the statistical significance of the observed differences between these groups, chi-square and Fisher exact tests were employed”

• Ethical Considerations (Line 12): The authors mention an ethics and regulatory board in their discussion on ethical considerations. Could they specify which board this refers to earlier in the text? The mention in lines 17 and 18 suggests a specific board, but clarity from the outset would help readers understand the ethical framework guiding the study. Additionally, considering this is a secondary analysis, could the authors clarify whether any form of Human subjects’ protection course or certification was completed by the research team? This information is crucial for readers to gauge the ethical rigor of your study.

RESPONSE: The authors have recorded that section to address this comment. Although the authors have completed the Good Clinical Practice course, however, the authors did not have access to non-anonymized data, so we think it may not be necessary to state our course completion for Human subjects protection.

• Results Section - Consideration of Rural vs. Urban Households: It is noted that 57% of the households in the study are in rural areas, leaving 43% in urban areas. This slight imbalance prompts a question regarding the potential impact on the statistical analysis. Have the authors considered applying weights to the data based on this rural-urban distribution? Although the current imbalance may not be significant, exploring this through weighted analysis could offer insights into whether it influences the results. This could be an interesting aspect to consider or discuss, even if only to validate the robustness of the findings.

RESPONSE: The authors fully agree with this, and would like to point the reviewer’s attention to the statement in the statistical procedures, “All results presented in the technical report were based on weighted estimates”. The authors have also included a statement in the discussion to buttress this.

**Reviewer #6**

Overall, the authors have rigorously followed and integrated all of peer reviewers observations, except mainly the following point :

Comment 3 : Since the data used in the research was a secondary data, line 22,23, 24 should be talking about data extraction (ie. How your variables of interest were extracted from the Nigeria HIV/AIDS survey dataset).

Authors response : The authors did not extract data from the Nigeria HIV/AIDS survey dataset – only the technical report of the survey was assessed and data was manually extracted from it.

My conclusion about this point : This response is acceptable. But, the authors must include this additional information in the manuscript as reported here: the technical report of the survey was assessed and data was manually extracted from it.

RESPONSE: The authors have adjusted the section based on this recommendation

3.2. My (review) comments

The manuscript provides a comprehensive overview of the HIV situation in Nigeria. However, in this manuscrit, hepatitis B and C are presented as opportunistic HIV infections (tables 2 and 3). We suggest these infections be considered as HIV co-infections. Regarding HIV opportunistic infections, we recommend to consider tuberculosis.

Unfortunately, this important study has not addressed HIV status among TB population due to the association exists between both infections. We know that TB is considered as the most common opportunistic infection among PLWH, and the prevalence HIV among TB population is often high particularly in African countries.

The same is true with the key populations (PS, MSM, IDUs, prisoners, etc.), whose prevalence is often higher than the national average in most African countries (Nigeria being no exception) and depending to the place of residence (rural/urban).

RESPONSE: The authors have adjusted this based on this recommendation on co-infection. However, we are unable to consider tuberculosis due to restriction based on available data. We have included this as a limitation in the limitation section, which future researchers can explore.

3.3. Recommandation :

It would be beneficial to address HIV status among TB population and among key populations (PS, MSM, IDUs, prisoners, etc.) based on the place of residence according to place of residence, as well as was done with the other groups in tables 2 and 3. Thus, if data are available, authors are invited to complete the tables 2 and 3 with the HIV situation in TB patients and in key populations. If data are not available, both situations should be discussed with evidence and references in the manuscript.

RESPOSNE: The authors think that discussing HIV status among TB population and key population may go well beyond our results scope, and think it is a research gap that should be considered in future studies.
