# Supplementary material for: On characterizing gender and locational composition of adult PLHIV in Nigeria: Implications for HIV programming
Source: PLOS Glob Public Health. 2024 Aug 26;4(8):e0002863. doi: 10.1371/journal.pgph.0002863 (PMC11346663; doi:10.1371/journal.pgph.0002863)
Supplement: Attachment — Submitted filename: Review Comments_08042024.docx [file pgph.0002863.s007.docx]

**Reviewer #3**

The ethical considerations, as well as the detailed interpretation of figures 2 and 3, were not addressed in this version. However, there was some discussion regarding the implications of observed regional and gender disparities, albeit only partially addressing how these insights can inform targeted interventions. This suggests a need for addressing ethical consideration comments in the first review as well as a deeper dive into the significance of the data presented in figures 2 and 3 to enhance the understanding of potential interventions aimed at addressing disparities.

RESPONSE: The authors appreciate this comment but will like to call the attention of the reviewer to the prose directly under Table 3 in the result section, and the second paragraph of the discussion section.

**Reviewer #5**

All my comments have been addressed sufficiently and the manuscript is now in a better shape and format for acceptance.

RESPONSE: The authors are immensely grateful for the reviewer’s comments and commitment to a publishable manuscript.

**Reviewer #7: Summary**

The authors have demonstrated a good understanding of the subject matter. Conclusions have also been reached covering key areas of the study objective. However, based on the interest of the study which essentially examined the distribution of HIV viz-a-viz its prevalence, diagnosis etc. across gender and location, analyses were basically descriptive and these have been well established. This limits the contribution of the study to literature. However, extending the study focus to examining causative factors particularly in respect of contexts/locations can improve the quality of the study.

Recommendation

Publish with major revision

Review feedback

RESPONSE: We appreciate this comment but would love to state our limitation to examining causative factors in this study. First, this study was cross-sectional in nature, and therefore cannot establish causality. Secondly, we established association with bivariate analysis and due to the fact that the study was limited to the availability of data – which was extracted from the NAIIS report, thereby limitinf the possibility of a multivariable analysis.

Page 2

Lines 7/11: Authors omitted sample size in the method section

Line 9: “These groups”: Authors may want to first, clearly identify the different groups

Lines 13/22: Evidence of statistical significance of difference between rural and urban not shown

Overall, it appeared the only location of focus, as shown in the abstract, was the rural/urban dichotomy. Such finding appear evident in literature already

RESPONSE: The authors have reworded to capture “these groups”. Furthermore, the authors would love to call the attention of the reviewer to the bivariate (chi-square test of differences) in Tables 2 and 3 that shows the evidence of statistical significance at 10%.

Additionally, the abstract did not only focus on the rural and urban differences, but also on the gender differences. The authors agree that this is evident in literature, but these findings are limited in resource limited countries like Nigeria. Similarly, this is one of the first studies to characterize the differences in Nigeria on a national scale.

Page 3

Lines 11/13: Authors may want to break sentences to smaller parts to improve clarity on the origin of HIV, its prevalence in sub-Saharan Africa and the current situation in the sub-region

RESPONSE: The authors are grateful of this comment, and have ensured that the manuscript is written as such.

Page 5

Lines 3/8: The paragraph appeared to have addressed the core focus of the study.

Lines 9/17: The strength of the study appeared to be the combined effect of gender and location as well as the use of a nationally representative study. However, the analytic method adopted appeared to be insufficient to address the objective. In particular, gender disparities among PLHIV have been well established, perhaps a focus on locational clustering and extension of analysis beyond bivariate level can further strengthen the justification for the study.

RESPONSE: The authors appreciate this comment but are limited in data availability to carry out a spatial analysis. We only attempted to characterize the gender and rural-urban differences.

Page 6

Line 5: While the sampling procedure for the survey was described extensively, specific study sample size was omitted by the Authors.

Line 11: Perhaps a justification for selected age range is essential

Page 7

Lines 20/23: Nice attempt but I wonder if more accuracy of sample can be achieved using the actual raw survey data, rather than the estimates derived from reported percentages and total sample size

RESPONSE: The age range used in this study consisted of individuals that are defined as “adults” according to the NAIIS survey. It is believed that the burden and transmission of HIV is higher within the 15-64 age group. Based on this comment/suggestion, the authors have kick-started efforts to obtain the actual survey data from NAIIS, and hopes for a positive feedback to make more meaningful contributes to literature on HIV.

Page 8

Line 6: A 10% significant level was used, unless of course there are justifiable arguments, the robustness of the analysis to detect true significance is reduced. The chances of committing a type 1 error is increased such that a true null hypothesis is rejected. Wrong decision might have been taken on the significance of differences in examining gender and location differences. I recommend testing the clustering at, at least, two different lower significant levels.

RESPONSE: We totally agree with this point and have reduced the level of significance to 5%.

Page 9

Line 1: Authors wrote 58% rural residents in the table but reported 57%

RESPONSE: We apologize for that error, and have updated this in the prose.

Line 7: Authors did not show sample size on Table 1. The practice is to either show the denominator (N) at the top of the table if the total sample for each variable in the table is the same or show total sample for each of the variable in the table

RESPONSE: We were constrained to including an overall sample size due to the differences in sample size across the variables. Previous reviews also recommended that we do not include the total sample size for each of the variables due to this.

Page 10

Significant levels of Table 2 were actually 0.1%, rather than the earlier 10% stated by the Authors

RESPONSE: We have updated this on the table, and reduced the significance to 5%.

**Reviewer #8**

The authors took into consideration the comments made by the reviewers, and made the appropriate corrections.

RESPONSE: Thank you
