# Supplementary material for: On characterizing gender and locational composition of adult PLHIV in Nigeria: Implications for HIV programming
Source: PLOS Glob Public Health. 2024 Aug 26;4(8):e0002863. doi: 10.1371/journal.pgph.0002863 (PMC11346663; doi:10.1371/journal.pgph.0002863)
Supplement: Attachment — Submitted filename: Review Comments_06062024.docx [file pgph.0002863.s008.docx]

**Reviewer #7**

INTRODUCTION

Page 4

9 2025, which is essential for the detection and prevention of mother-to-child transmission of

HIV (PMTCT) before pregnancy among reproductive age.

Response: The authors have edited this statement and rephrased it to “Another targeted HIV program in Nigeria is the achievement of 90% coverage of antiretroviral therapy (ART) among HIV positive women by the year 2025 - this is essential for the detection and prevention of mother to child transmission of HIV (PMTCT)”.

Page 7

2 “Prevalence of HIV” I think is reported in proportion, not frequency

Response: The prevalence of HIV was reported in both frequency and percentage (Table 2).

6 Self-reported HIV testing: Number of participants who REPORTED ever receivING an HIV test result and receivING an HIV test result. I presume the REPORTED part was omitted.

Response: The authors appreciate this comment, and have updated this.

RESULTS

Page 9

3 Rural population proportion is 58% as shown in Table 1 (This was not updated)

Response: The authors agree that this was an oversight and have updated the same.

Page 10

4 Chi square and Fisher exact tests are either testing for association or independence. Based on the objective of this manuscript, the tests are for association, not difference as reported across the manuscript. Reporting difference could suggest that tests of mean difference have been conducted. That can be misleading. I would advise that Authors report the distributional proportions. For example, rather than saying “there is a significant difference”, I would say ”there is a significant variation in the distribution…”

Response: The authors have updated this, as suggested.

5 Reporting p-values as being less than 0.001 suggests you are comparing your estimates’ p-values with 0.1% level of significance (These have not been updated)

Response: The values that the authors are trying to point out were “0.000” according to the statistical analysis. We have included a footnote to state the level of significance to indicate this.

DISCUSSION

Page 18

6 I would advise hat Authors begin the discussion section with the strength of this study. First few paragraphs should focus on discussing findings from this study (rather than those from NAIIS) and relate to findings from literature including NAIIS report.

Response: The authors have updated the discussion to reflect the study strength rather than NAIIS strength, as suggested by the reviewer
