# Supplementary material for: On characterizing gender and locational composition of adult PLHIV in Nigeria: Implications for HIV programming
Source: PLOS Glob Public Health. 2024 Aug 26;4(8):e0002863. doi: 10.1371/journal.pgph.0002863 (PMC11346663; doi:10.1371/journal.pgph.0002863)
Supplement: Attachment — Submitted filename: Response to reviewers_29072024.docx [file pgph.0002863.s009.docx]

**Comments**

Reviewer #7: Generally, while authors have improved the manuscript draft based on some suggestions earlier highlighted, other suggestions are yet to be accommodated. An instance is the significance level which still appears as though analysis was done using 10% significant level. I would implore authors to adopt “association” rather than “difference” used in the reporting. This can be misleading as Chi-square/Fishers’ tests used here are tests of association, not of difference. For example, report can be written as: “There is a significant association between location and HIV prevalence as higher prevalence was observed among rural residents compared to those in urban areas”.

RESPONSE: The authors can confirm categorically that there was no point in the paper where the significance level was set at 10%. Furthermore, earlier in the review process, a reviewer suggested that we change all “associations” to “differences” since we were quantifying the differences and not necessarily reporting associations. In essence, we aimed to determine “whether the difference between rural and urban is big enough to be statistically significant”.

Page 4

Lines 5-7: At the end of this sentence, “The aim is to guarantee that 90% …”, the “respectively” appears to suggest something is missing. While the two milestone dates were stated, the events to which they individually refer are not clear.

RESPONSE: The authors appreciate this feedback and have update the information to read “The aim is to guarantee that 90% of the population, including vulnerable and important populations, has access to HIV combination preventive programs by 2030”.

Page 9:

Update Table 1 where rural percent is 57% as written on Page 9, Line 3. I think the rural-urban divide percentages needs to be updated

RESPONSE: We appreciate the comment and would love to state that the information from the first paragraph refers to the entire household surveyed in the NAIIS. The last sentence contains information on the participants surveyed in this study.

In Tables 2 and 3, it appears that the authors still tested the associations between the outcomes and Gender and locational variable at 10% (0.001) rather than the 5%(0.05) earlier mentioned. This should be updated in both tables and corresponding reports. If these tests had been done at 5% level, it would be more appropriate to compare tests p-value to the significant level i.i p<0.05 rather than p<0.001.

RESPONSE: The authors followed the guidelines of PLOS GLOBAL PUBLIC HEALTH which states: “Report exact p-values for all values greater than or equal to 0.001. P-values less than 0.001 may be expressed as p < 0.001”. Furthermore, we would love to highlight that 10% implies 0.1 rather than 0.001.

Page 19:

Line 4: “reported by the as the …”, something appears to be missing in this sentence

RESPONSE: The authors appreciate the meticulous attention to detail by the reviewer. However, we think the reviewer read the version with tracked changes.

Page 22:

Lines 8-10: The strength of this study can be better represented if specific limitations can be mentioned rather than the generic sample size, sampling methodology and measurement validity mentioned. NAIIS is a nationally representative data which incorporated rigorous method design, the extent to which earlier mentioned limitations affect representativeness of this study and its generalizability should be stated and clarified.

RESPONSE: The authors have updated the strength and limitation section of the study.
